# Study on formation mechanism of mud-inclusion-type underground debris flows using natural caving method

**DOI:** 10.1038/s41598-024-54082-0

**Published:** 2024-02-21

**Authors:** Xiangdong Niu, Kepeng Hou, Guangtuo Bao, Yalei Zhe

**Affiliations:** 1 Faculty of Land and Resources Engineering, Kunming University of Science and Technology, Kunming, 650093 China; 2 Yunnan Key Laboratory of Sino-German Blue Mining and Utilization of Special Underground Space, Kunming, 650093 China; 3 Yunnan Yarong Mining Technology Co., Ltd., Kunming, 650000 China; 4 Kunming Metallurgy College, Kunming, 650033 China

**Keywords:** Natural caving method, Mud inclusion, Underground debris flow, Generalized model of mechanism, OCTEM method, Experimental study, Environmental sciences, Natural hazards

## Abstract

This study aimed to investigate the formation mechanism of the mud-inclusion-type underground debris flows of natural caving underground mines. The characteristics of fine moraine particles flowing through the coarse-grained ore bed were used to analyze the formation process of mud inclusions in the caving ore bed through a physical model test. Based on the movement behavior of the mud inclusions of moraine in the caving ore bed, a formation-mechanism generalized model of underground debris flows with mud inclusions was established. The model was used to examine the formation mechanism of mud-inclusion-type underground debris flows in natural caving. The results showed that the fine moraine particles had good cross-flow characteristics in the process of drawing coarse-grained ore. The accumulation of fine moraine in the ore bed was a prerequisite for the formation of mud inclusions, and the fluid inclusions were formed by a mixture of the particles with the infiltrated water. When mud inclusions in moraine are affected by many factors, such as ore-drawing vibrations, blasting vibrations, and groundwater, the inclusions undergo multiple migration–stop–migration cycles, resulting in separation or fusion. However, the inclusions are released along the optimal random pore path to the outlet, forming a certain scale of underground debris flows accidents. The accuracy and reliability of the formation mechanism were verified through geophysical explorations based on the equivalent inverse flux transient electromagnetic method. This study not only broadens the research on debris flow, but also provides theoretical guidance for the prevention and control of underground debris flows.

## Introduction

Natural caving is a large-scale underground mining method, which can be used for high-efficiency large-scale mining. It is also known as the ore block caving method, whereby natural stress is taken as the main load of the ore body. Natural caving is the only mining method that is comparable to open-pit mining in terms of production costs. Compared with other underground mining methods, the natural caving method has the following advantages: simple underground stope layout, high mechanization, large production capacity, low mining cost, good economic benefits, and so on. The mining method is especially suitable for the mining of super-large, low-grade ore bodies, and it can improve the utilization ratio of underground mineral resources. The natural caving mining method has been widely applied in several countries, and its application in China is becoming more widespread. Certain caving mines have fine moraines or granular materials on the ground surface. The upper overburden of an ore body contains small particles, and the existence of fine particles in the overburden causes unusual disasters during mine production. For example, the Pulang Copper Mine is a natural caving mine with fine moraines on the surface, and the open pit-to-underground caving mine isolates the fine particles of the overburden layer on the ore pillar. During ore drawing, fine particles flow through the loose pores of the caving ore bed^1–3^. Consequently, the ore dilutes prematurely, which easily induces underground debris flows during the rainy season, creating a significant threat to the production safety and economic benefits of the mine^4,5^.

Essential differences exist between the occurrence mechanism of mine debris flows and natural surface landslides, such as debris flows and tunnel debris flows^6,7^. Currently, research on the mine debris flow mechanism focuses on the slope of open-pit mine dumps. However, the mechanism of underground debris flows has received little research attention^8–12^, where the existing literature contains only a brief introduction to underground debris flows, a simple occurrence mechanism, and prevention measures. Wang et al.^13,14^ studied the debris flow in Jinshandian iron mine using physical similarity tests. By inference, the authors determined that underground debris flows occur in the rainy season and mostly in the ore sections containing fine particles. Furthermore, they noted that the overhanging roof of the fine-particle ore section is an effective focus area for preventing underground debris flows. Liu et al.^15^ used the physical similarity model test method to study the underground fine-particle-ore debris flow in Jinshandian iron mine. The results showed that water pressure increased as mining and production advanced. Mining activity and production vibrations cause vibrations in the key blocks in critical equilibrium, inducing underground debris flow accidents. Chen et al.^16^ studied the mechanism of water and sand inrush under different loose overburdens. The results showed that 5% and 10% clay contents in loose overburden promote water accumulation and sand bursts. Compared with the case of a 5% clay content, the possibility of water and sand inrush reduces at a clay content of 10%. When the clay content is 20%, the sand body remains stable, restraining the inrush of water and sand.

According to the authors’ current research results, underground debris flows in an underground mine can be divided into two types: mine drawing-channel-type underground debris flows and mud-inclusion-type underground debris flows^17,18^. The sudden and hidden nature of the mud-inclusion-type underground debris flow poses serious difficulty in underground mine research. The indoor physical model test method provides reproduction, clear visualization, and reliable modeling for the phenomenon; thus, it has been used in debris flow research. However, the method is limited by its long test cycle, high working intensity, and high economic cost. Currently, the indoor physical similarity simulation experiment of underground debris flows has provided fewer key research results, hindering advances in underground debris flow research^19–21^.

At present, the physical model method is mainly used to study underground debris flows. However, the results of such research have the following shortcomings: first, the research methods and techniques are relatively not diverse, and their accuracy is low. Second, the research on the formation mechanism of underground debris flows has not been sufficiently in-depth, and its proposed simple mechanism is only a superficial introduction of the phenomenon. Hence, the coupling relationship between its main influencing factors has not been studied intensively. Moreover, compared with open-pit mine debris flows, underground debris flows have a more complex formation mechanism, which depends on the complex stratum structure, changeable stope boundary conditions, and influence of many mining factors. Potential goafs of different sizes and shapes also exist in the surrounding rock of the stope. Meanwhile, the mechanical properties, as well as deformation and failure mechanisms of the medium, dynamically change with space and time under the influence of complex and changeable mining factors. Therefore, little progress has been made in the research on the mechanism, prevention, and control of underground debris flow disasters induced by natural caving. The formation mechanism of underground debris flows with mud inclusion was investigated in this study through an indoor physical model test. The study provides a basic theory for the prevention and control of underground debris flows.

## Physical model test design

### General condition of mining area

**Figure 1:**
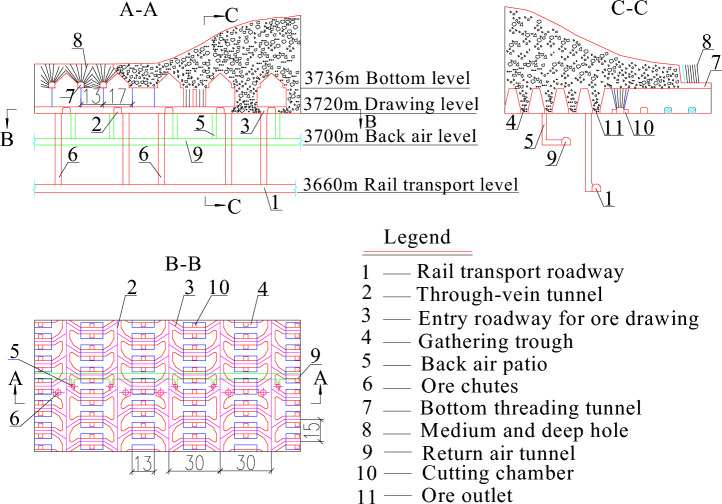
Mining method map of the Pulang Copper Mine.

The Pulang Copper Mine, which is owned by Yunnan Diqing Non-ferrous Metal Co., Ltd. (located in Shangri-la, China), is mined using the natural caving method; see Fig. 1. The annual mining production scale is 12 million tons. The tectonic activity around the Pulang Copper Mine is strong, accompanied by the development of faults, secondary folds, and joint fissures. The main exposed strata in the mining area are the second member (Tn2) of the Middle Triassic niru formation, the Upper Triassic tumou formation (T3t), and the local exposed Quaternary (Q). The strata in the mining area comprise both new and old strata; their thickness parameters are as presented in Table 1.

**Table 1:** The stratigraphic table of Pulang Copper Mine.

Stratigraphic system						Stratum code	Thickness
Erathem	System	Series	Formation	Member	Stratum		
Cenozoic	Quaternary system					Q	0–81.59
Mesozoic Erathem	Triassic system	Pliocene series	Tumou formation	Second pragraph	Second stratum	T_3_*t*^2–2^	> 1000
					First stratum	T_3_*t*^2–1^	1000
				First paragraph		T_3_*t*^1^	> 400
		Mliocene series	Niru formation	Second paragraph		T*n*^2^	> 1485.2

### Similarity theory of physical model test

The similarity theory is the basis for the physical model test, which aims to determine the internal law of the model at the lowest cost and the shortest period. In the similarity model test, the prototype is scaled into the model at a certain proportion. This test generally needs to satisfy the geometric, kinematics, and dynamics similarities, as well as the material or medium physics similarity^22–24^. For physical model tests, geometric similarity is the first similarity principle to be followed, and it is also the easiest similarity condition to adjust technically. Precisely, the geometric similarity means that the size of the prototype is proportional to the size of the model. Meanwhile, the kinematic similarity requires the motion laws of the physical model and the corresponding points in the prototype to be similar, while a certain proportion is maintained for the motion time.

### Test models and media

**Figure 2:**
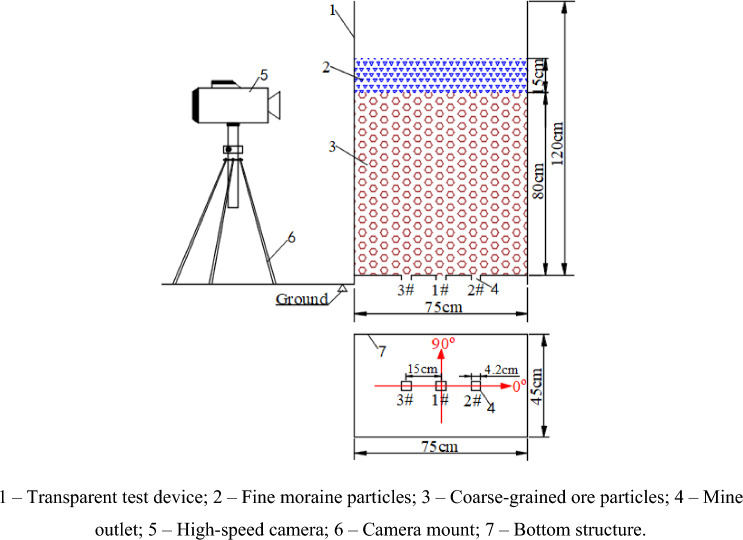
Design of 3D indoor physical model test device.

According to the related technical parameters of the natural caving method of Pulang Copper Mine, the distance between the center and the adjacent drawing holes is 15 m, and the drawing hole size is 4.2 m × 4.0 m. According to the similarity theory, and using a geometric similarity ratio of 1:100, the drawing hole size of the indoor drawing model is 4.2 cm × 4.0 cm, and the distance between the center and the drawing hole is 15 cm (see Fig. 2). The indoor physical model test device used in this study was mainly composed of a drawing box, bottom structure, and camera system, as illustrated in Fig. 2. The size of the drawing box (length × width × height) was 75 cm × 45 cm × 120 cm. To observe the flow of fine particles and the formation of mud inclusions, the drawing box was made of transparent acrylic sheets. The bottom structure had three ore-drawing openings: 1#, 2#, and 3#. The dimension of the drawing hole was 4.2 cm × 4.0 cm, and the distance between the drawing holes was 15 cm.

**Figure 3:**
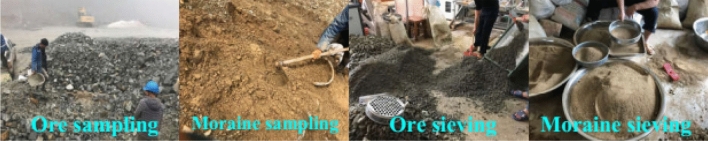
Field sampling map of ore and moraine.

**Figure 4:**
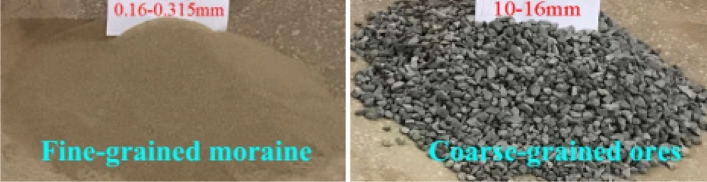
Test media of fine-grained moraine and coarse-grained ore.

**Figure 5:**
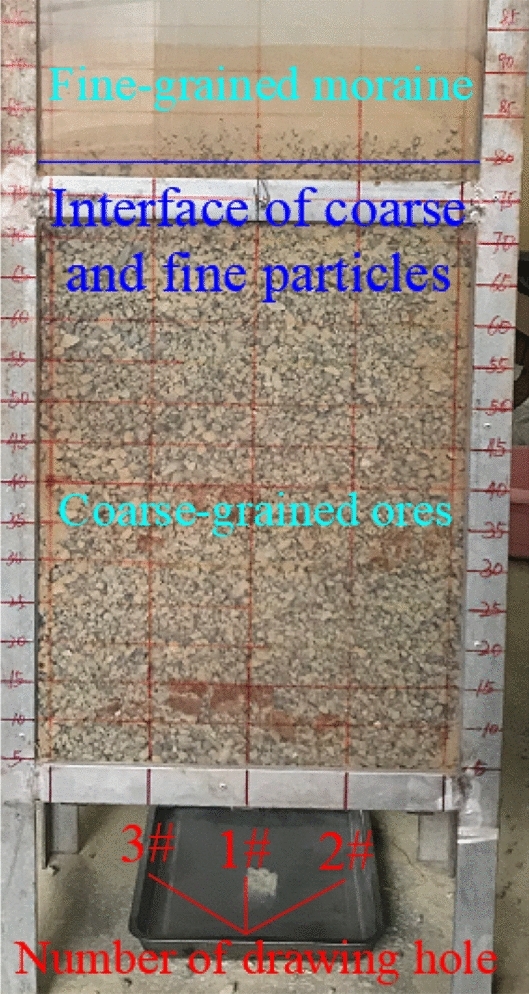
Material test model after filling.

Both the coarse-grained ore and fine-grained moraine particles used in the tests were collected from the Pulang Copper Mine. Figure 3 shows personnel at the mine site performing sampling. Standard Sieve (JGJ52-2006) was used to screen the particulate matter used in the experiment. The particle size of fine moraine was 0.160–0.315 mm, and the diameter of the coarse-grained ore was 10–16 mm, as shown in Fig. 4. The coarse-grained ore was heaped at the bottom of the physical model up to a height of 80 cm, and the fine moraine was loaded on the coarse ore up to a height of 15 cm. The material test model after filling is shown in Fig. 5 and was used to simulate the ore drawing conditions under the Quaternary fine-grain overburden on the caving ore bed.

### Physical test hypothesis

The movement of moraine and ore particles in mediums was highly complex during the drawing test of the physical model. To avoid the influence of other factors on the test results, some influencing factors were considered, and the following assumptions were made: (1) for good fluidity of the medium during the test, the moraine and ore particles were air-dried; hence, no adhesion existed between the particles. (2) Owing to the high particle strength of the ore, the effect of secondary fracture during the uniform ore drawing was negligible. (3) The effect of artificial loading was negligible when the test particles were filled using the central quadrant method.

### Analysis of test results

In the test drawing model, the ore was drawn in turn through the orifices (1# → 2# → 3#) until all the particulate matter was released from the model device. To simulate a uniform ore-drawing environment, the discharge quantity of each drawing hole was the same. Moving through the three ore-drawing orifices was one drawing cycle, and four drawing cycles were performed in the entire test process.

**Figure 6:**
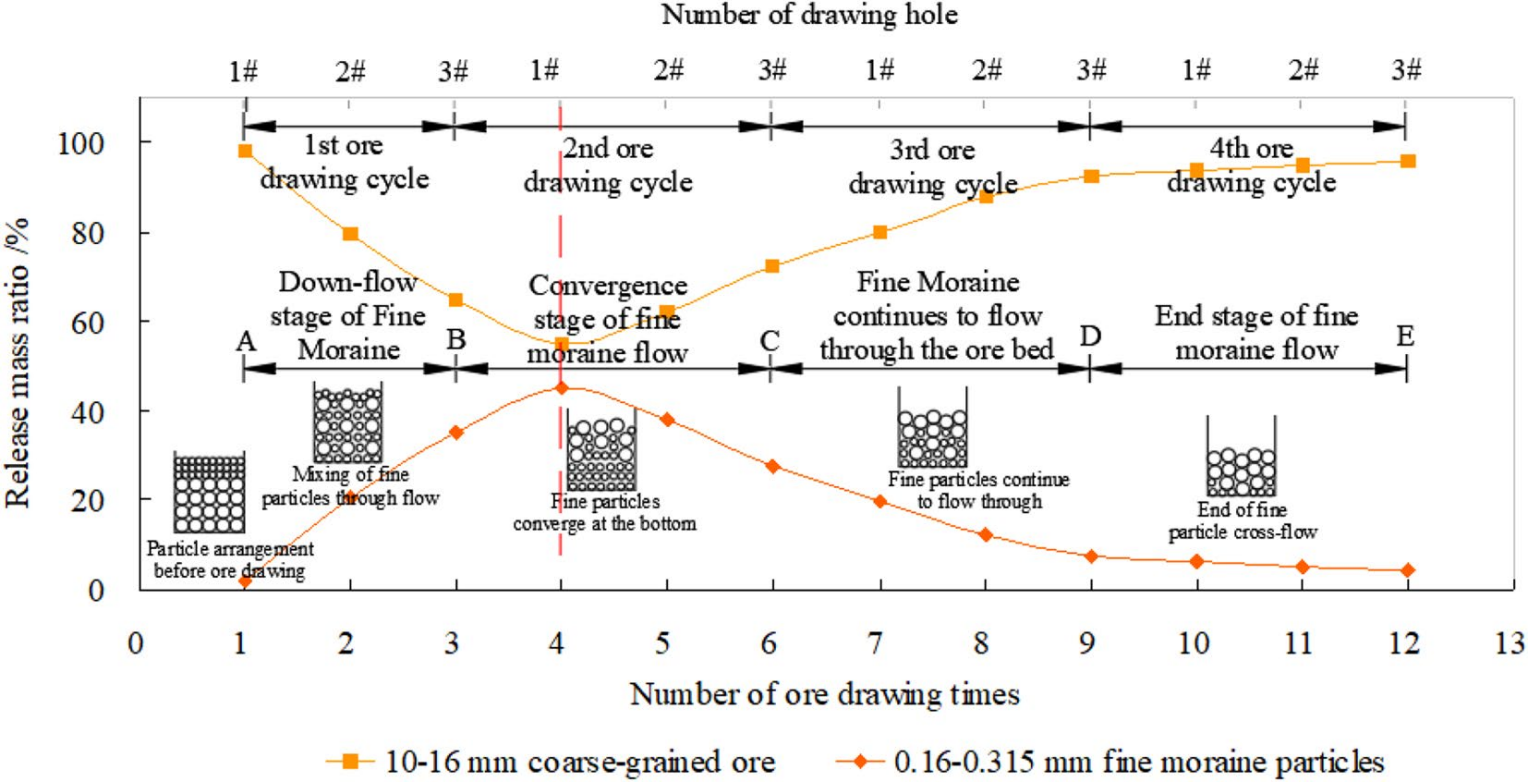
Mass ratio curve of current release of fine-grained moraine and coarse-grained ore.

A standard sieve was used to screen the discharged particulate matter immediately after each drawing. The weights of fine moraine and coarse ore were obtained. The “normalization method” was used to process the test data, and the mass proportion of fine moraine and coarse ore released at the same time was obtained, respectively^25,26^. Figure 6 illustrates the relationship between the fine moraine and coarse ore discharge proportions, as well as the drawing times and cycles.

Figure 6 shows that the proportion of fine moraine particles released at proper times increased first before decreasing as the ore-drawing time increased. Hence, the fine moraine had a highly significant effect on the flow through the coarse ore during ore drawing. The fine moraine particles moved downward prior to the coarse moraine particles and accumulated in the coarse moraine layers. This phenomenon provides favorable material source conditions for the formation of mud inclusions during the rainy season.

## Mechanism of mud inclusion type underground debris flows

### Formation of mud inclusions

**Figure 7:**
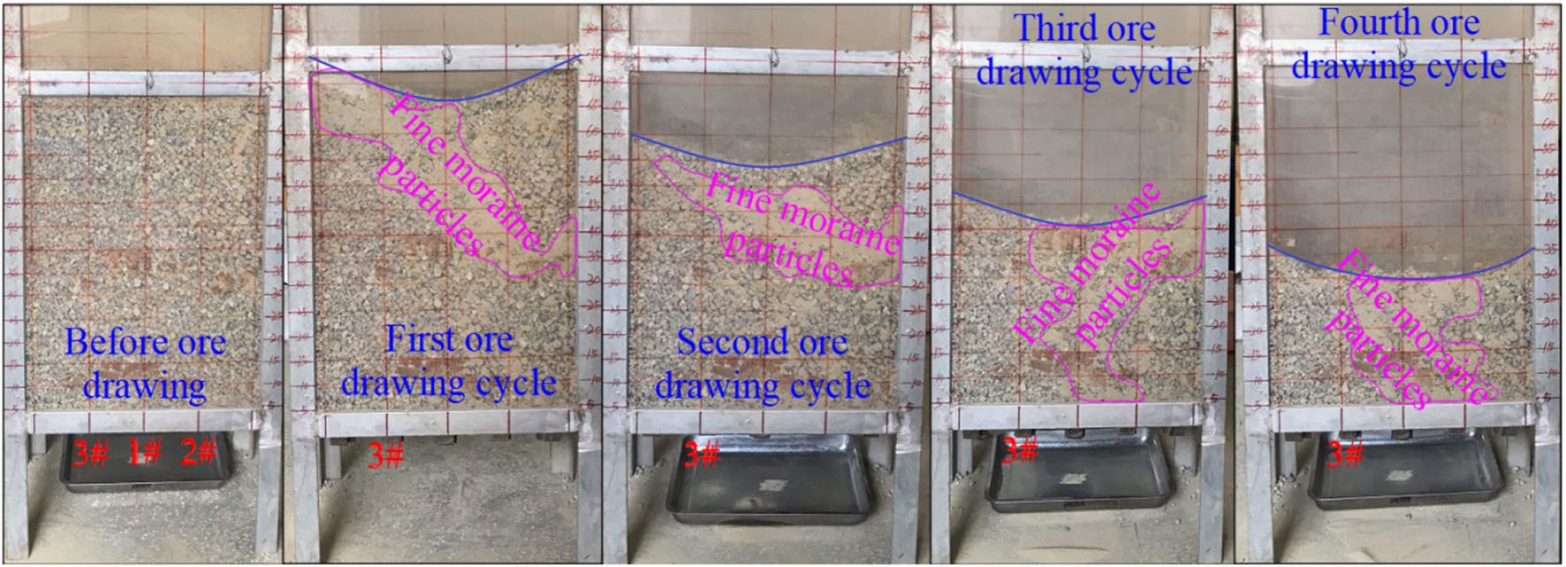
Motion regularity of fine-grained moraine during uniform ore drawing.

The physical model test results indicate that the fine moraine had a downward movement during the drawing of the coarse-grained ore. The fine moraine with a cross-flow movement was concentrated in the ore bed, which provides favorable conditions for the formation of mud inclusions. In addition, from the “drawing theory” perspective, cross-flow behavior occurs when the diameter of a fine-particle medium is one-third to half of the pore size of the coarse particle medium^27–30^. Figure 7 shows part of the physical model test phenomena of the macroscopic cross-flow of fine moraine particles. As observed from the figure, the fine moraine particles gradually moved down and accumulated in the ore bed during ore drawing. Additionally, the fine moraine of 0.160–0.315 mm exhibited good flow characteristics in the coarse-grain ore bed of 10–16 mm. These conditions are favorable for the formation of mud inclusions in the caving ore bed.

**Figure 8:**
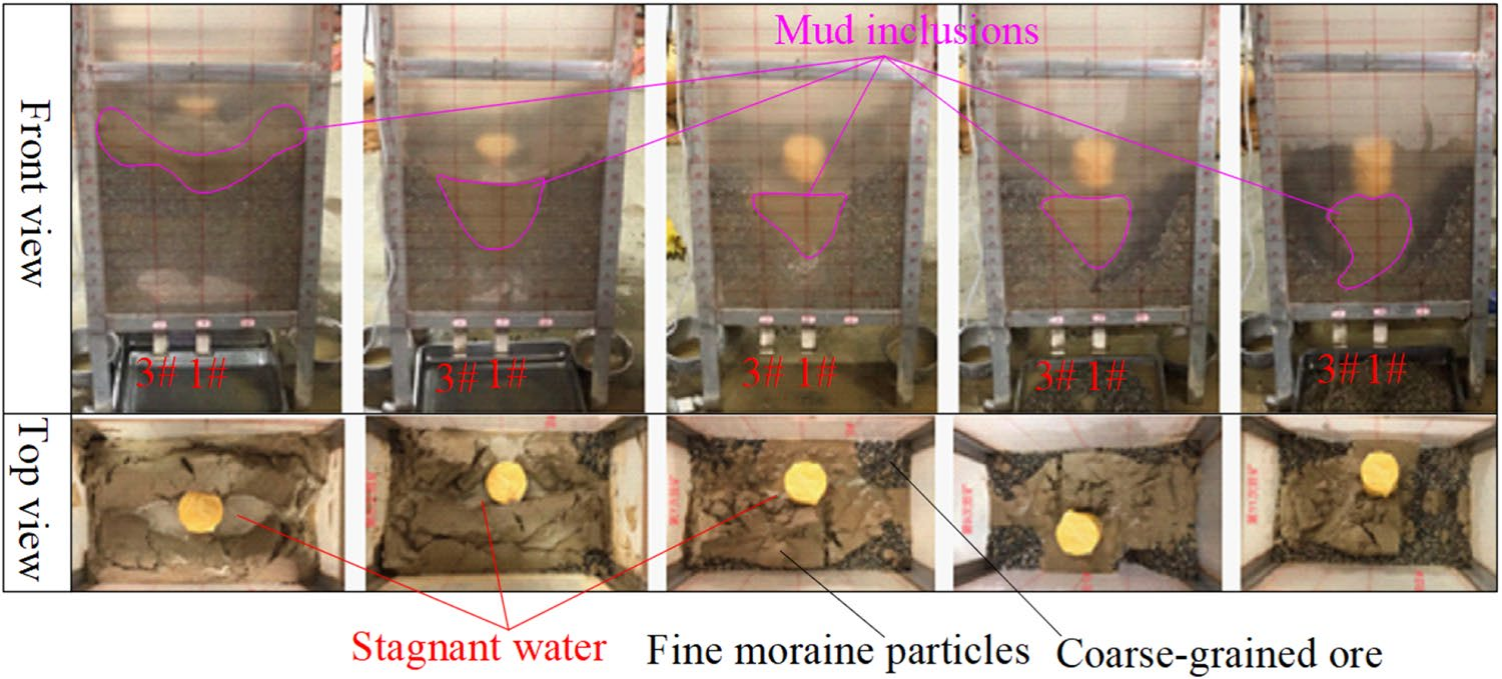
Schematic representation of mud inclusions in collapsed ore bed.

The analysis shows that fine moraine has good flow-through characteristics during coarse-ore drawing. As the ore is released, the flow-through movement occurs continuously, causing the fine moraine to gather continuously in the ore layer. Thus, favorable material source conditions are provided for the formation of mud inclusions. The rainy season is from June to October at the Pulang Copper Mine. The rain lasts long and is concentrated. Hence, rainfall and surface runoff in the mining area flow into the sinkhole and continuously penetrate the mine, mixing with the fine moraine accumulated by the cross-flow movement. Finally, a fluid-prone mud inclusion is formed in the caving ore bed, as shown in Fig. 8.

### Analysis of formation mechanism

**Figure 9:**
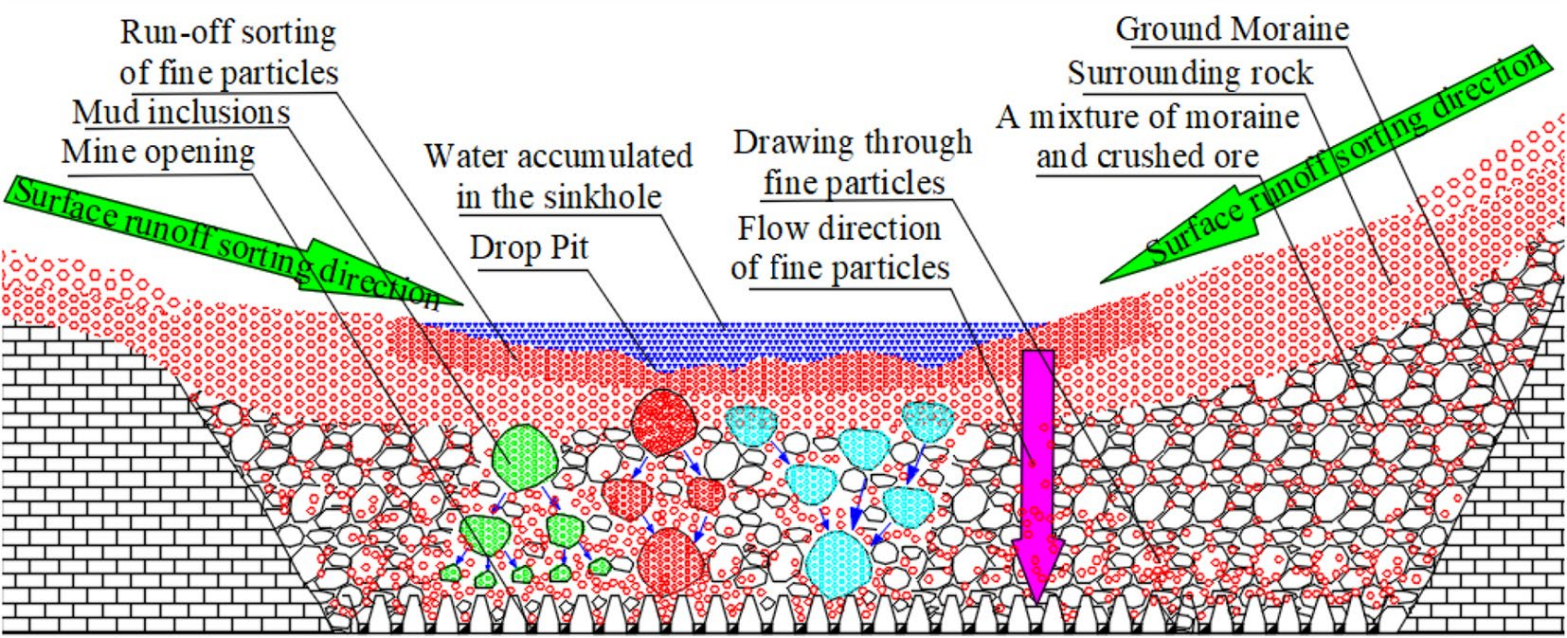
Generalized model of formation mechanism of mud-inclusion-type underground debris flows.

Because of the cross-flow characteristics of fine moraine particles, the particles precede coarse ore in downward particle movements and gather in the ore layer. The particles finally combine with the infiltration of rainwater to form easy-to-flow mud inclusions. During the continuous, daily ore-drawing cycle, the state of moraine mud inclusions is always in the “migration–stop–migration” cycle. Meanwhile, mud inclusions of moraine may be separated or fused when they are affected by mining vibrations, blasting vibrations, groundwater, and other factors. However, the “inclusions” are eventually released along the optimal random pore path to the outlet, and a certain scale of underground debris flow accident occurs. The formation mechanism of mud-inclusion-type underground debris flows is illustrated in Fig. 9.

**Figure 10:**
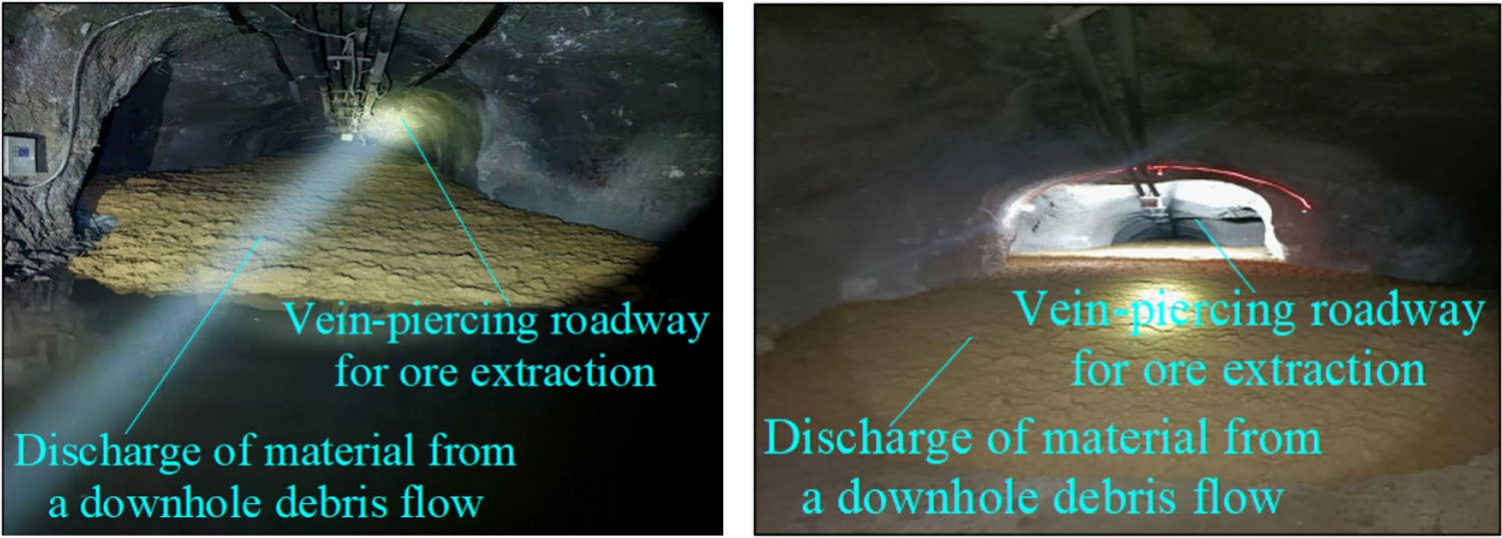
Field map of underground debris flows with mud inclusions in Pulang Copper Mine.

The underground debris flow material has a certain cohesion because its water content is smaller than that of the mine-drawing-channel type and the consistency of moraine mud inclusions is larger. Hence, the underground debris flow type is called the mud inclusion type. The material state of the underground debris flow in the Pulang Copper Mine is shown in Fig. 10.

### Verification of mechanism generalization model

From the year 2019 to 2022, more than 10 underground debris flow accidents occurred in the Pulang Copper Mine. The mud inclusion type of underground debris flows occurred four times, and the scale of each occurrence was less than 500 m^3^. The statistical information of the mud inclusion type of underground debris flows in the Pulang Copper Mine is shown in Table 2.

**Table 2:** Statistical information of mud-inclusion-type underground debris flows in Pulang Copper Mine.

Date of occurrence	Time of occurrence	Mine outlet of occurrence	Debris flow volume (m^3^)	Influencing factors of debris flow occurrence
2019.8.18	00:20	S7-E13	280	The next outlet is being mined
2019.8.20	21:10	S6-E11	120	The next outlet is being mined
2019.9.9	23:00	S8-E14	500	–
2019.10.11	22:30	S7-E18, S8-E13	500	–

**Figure 11:**
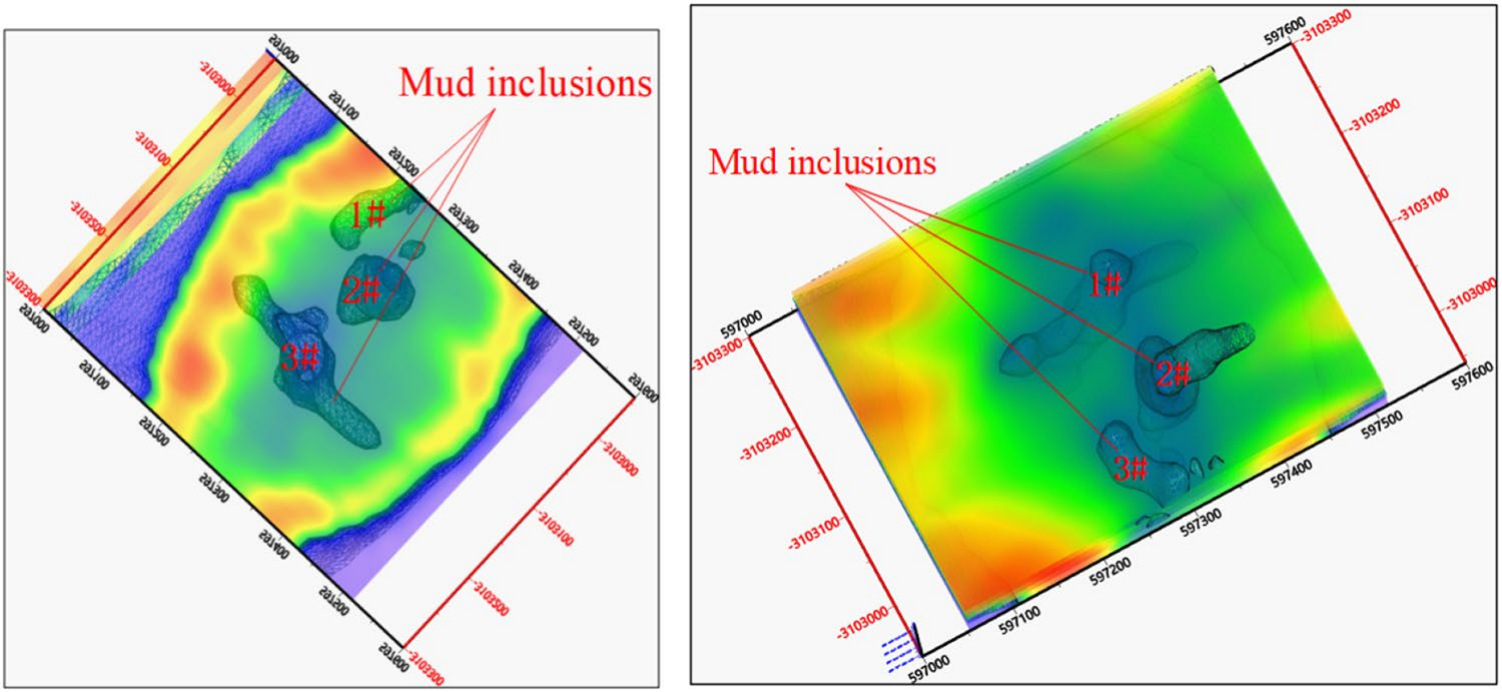
Spatial distribution and size of “mud inclusions” detected through OCTEM method.

Currently, many kinds of advanced detection methods exist for geological anomaly, such as geological, advanced pilot pit, and geophysical exploration methods. The geophysical prospecting method involves detecting the geological body indirectly using instruments, and the geological structure is not destroyed in this process^31–33^. The equivalent anti-flux transient electromagnetic method (OCTEM) is a fast, non-destructive, and strong anti-interference transient electromagnetic method. It can be used to detect the obvious low-resistivity anomaly area (“mud inclusion” of water-bearing moraine) in the caving ore bed. The distribution and size of mud inclusions with a certain scale can also be explored using the method, and the spatial location of mud inclusions in the caving ore bed can be located^34–36^. Therefore, we used the method for detection and to validate the formation-mechanism generalized model of mud-inclusion-type underground debris flows in the Pulang Copper Mine. The spatial distribution and size of mud inclusions in the moraine detected through the OCTEM method are illustrated in Fig. 11.

As shown in Fig. 11, many moraine mud inclusions were distributed in the caving ore-bed space of the Pulang Copper Mine. The effect of many factors, such as mining vibration, blasting vibration, and groundwater, resulted in separation or fusion, as well as variations in shape and scale. During the rainy season, numerous moraine mud inclusions with different shapes were formed in the caving ore bed. With continuous production, the moraine mud inclusions reached the outlet along the optimal random pore path and were released, forming a certain scale of underground debris flow accidents.

The OCTEM geophysical prospecting method was used to detect moraine mud inclusions in the caving ore bed of the Pulang Copper Mine. The existence of numerous moraine mud inclusions in the space of the caving ore bed also verify the accuracy and reliability of the study on the formation mechanism of this type of underground debris flow.

## Prevention and control measures of underground debris flows

### Prevention and control measures

**Figure 12:**
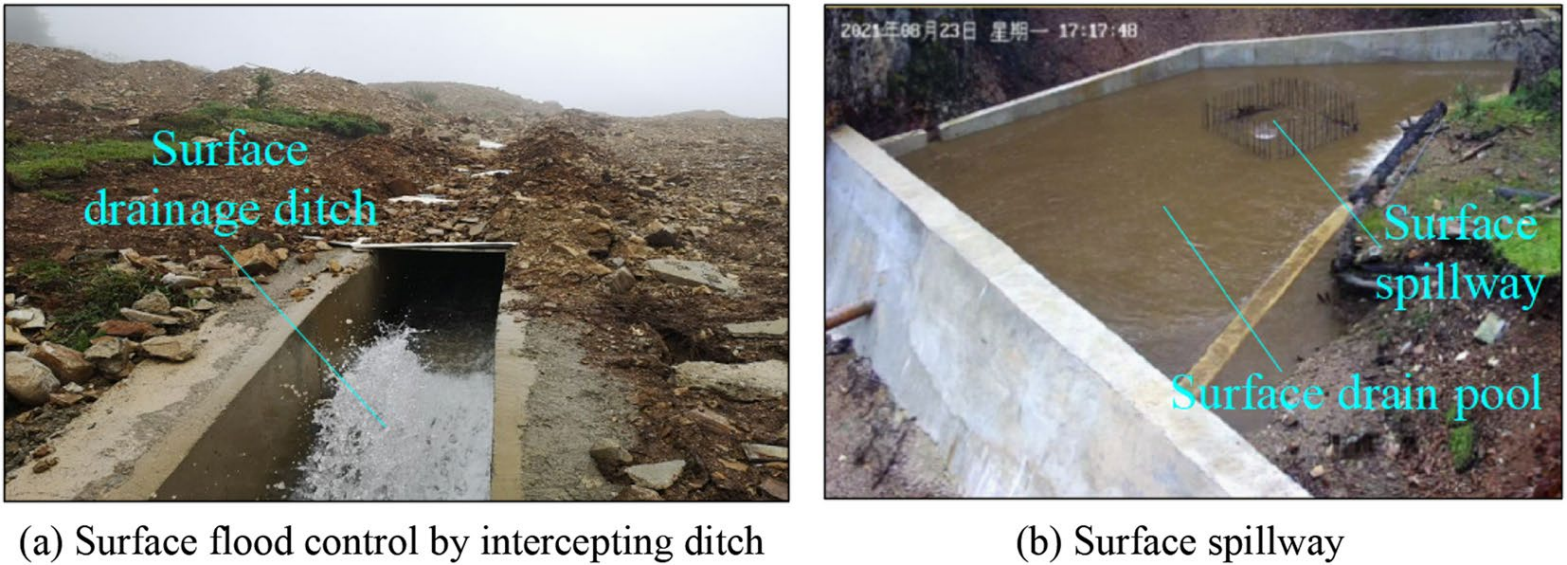
Surface flood control and drainage system within the Pulang Mine sinkhole.

**Figure 13:**
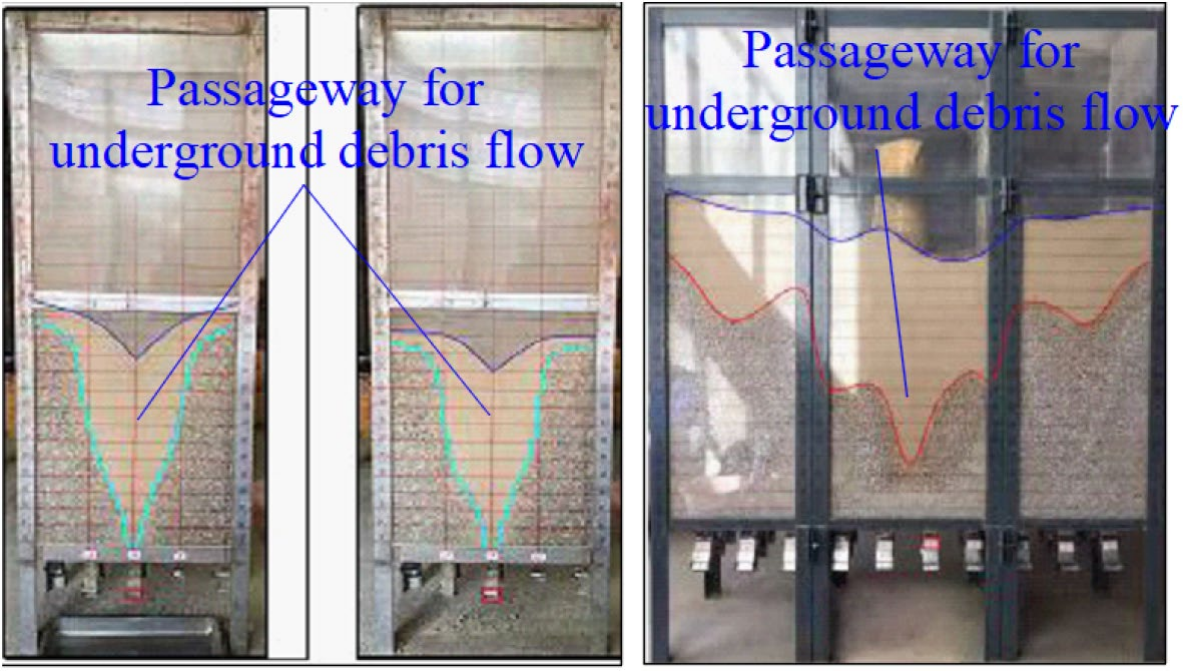
Moraine logistics channel under indoor unbalanced ore drawing conditions.

For the natural caving accident, the results obtained from studying the inducing mechanism of underground debris flows provide guidance for controlling underground debris flows. Thus, the following measures can be taken:

1. *Prevention and control measures of material source conditions*: In view of the landslide material source in the surface subsidence pit, some measures such as the slope body reinforcement can be adopted to prevent the slope body from sliding down and the fine moraine material from entering the subsidence. This can help to avoid the formation of an underground debris flow due to the material source conditions.
2. *Prevention and control measures for hydraulic conditions*: Water is an important factor in the occurrence of a debris flow. Water enters into the mine subsidence pit in two primary ways: atmospheric rainfall around the subsidence pit and surface runoff of rainfall around the subsidence pit. Water mainly enters the sinkhole through surface runoff. Therefore, the hydraulic conditions for preventing and controlling surface runoff include “interception, drainage, blocking” and other comprehensive prevention and control measures. The surface runoff of rainwater outside the sinkhole is collected and channeled outside the sinkhole for discharge. In this way, the surface runoff of rainwater upstream of the sinkhole is effectively intercepted, preventing it from entering the sinkhole as much as possible, as shown in Fig. 12.
3. *Prevention and control measures for channel conditions*: When the fine moraine particles overlying the ore layer undergo uneven drawing, a moraine flow channel is easily formed in the caving ore layer, as shown in Fig. 13. If the debris flow channel is formed in the caving ore bed for various reasons during ore drawing, the channel can be blocked by adjusting the ore drawing mode.

### Evaluation of prevention and control effect

**Figure 14:**
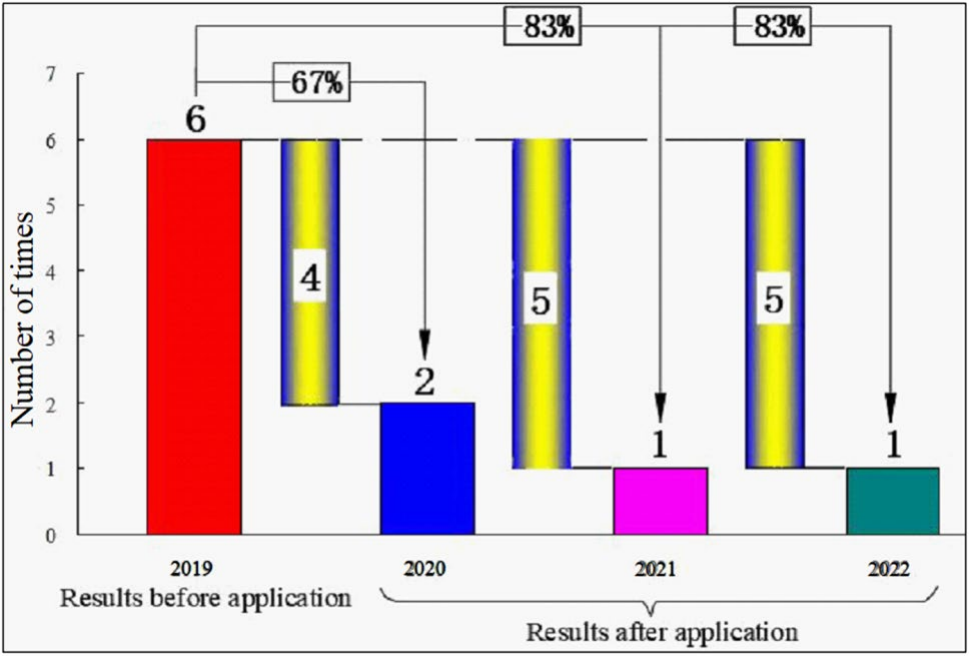
Frequency and reduction rate of underground debris flow in Pulang Copper Mine from 2019 to 2022.

In 2019, the Pulang copper mine started experiencing underground debris flow accidents, with six accidents recorded. The author conducted a systematic study on the formation mechanism and measures for preventing and controlling the underground debris flow accidents in the mine. Since 2020, the research results of the Evaluation Standard and the division of prevention and control of underground debris flows have been applied to prevent and control underground debris flows in the mine, deepening the understanding of the phenomenon. The mine also encountered underground debris flow accidents in both 2021 and 2022. Figure 14 illustrates the frequency and reduction rate of underground debris flows in the Pulang Copper Mine during the rainy season from 2019 to 2022.

According to Fig. 14, the method proposed in this paper for preventing and controlling underground debris flows has been successful for such flows caused by natural caving. Additionally, the number of underground debris flows in the Pulang mine decreased considerably in the past years, which also verifies the correctness and reliability of the developed approach to preventing and controlling underground debris flows caused by natural caving.

## Discussions and limitations

### Discussions

Natural caving is a mining method with high mechanization, good safety, high production efficiency, and low cost. It is the only low-cost downhole mining method comparable to an open pit. Currently, natural caving is widely used in several countries, but the domestic application has also shown a rising trend. During natural caving, the continuous extraction of underground mineral resources gradually expands the underground space, which inevitably leads to surface subsidence deformation and subsidence. As the mining depth increases, more frequent debris flow disasters occur with continued mining. Therefore, the results of this study can provide guidance for the accurate prevention and control of underground debris flows. Moreover, this study not only broadens the research on debris flow in general, but also provides theoretical guidance for the prevention and control of underground debris flows.

### Limitations

At present, there is limited research on the natural caving method, which makes it difficult to study the inducing mechanism of natural caving. In addition, many factors affect the formation of underground debris flows in a natural caving mine, and certain interactions and coupling effects exist among these factors, hindering research on the formation mechanism of underground debris flows. Owing to the limitation of current particle system testing techniques, the interaction forces between particles and the effect of drawing shear force on underground debris flow were not considered in this study. Therefore, in the future, advancements in science and technology will enable the author to improve studies on the inducing mechanism of underground debris flows.

## Conclusions

In this study, the characteristics of fine moraine particles flowing through the coarse-grained ore bed were studied using the indoor physical model test method. The formation process of mud inclusions in the caving ore bed was analyzed. The movement behavior of moraine mud inclusions in the caving ore bed indicated the formation mechanism of the mud inclusions in natural caving. The findings were verified using a geophysical prospecting method. The main conclusions are as follows:

1. According to the results of the physical model test and from a “drawing theory” perspective, fine moraine particles have good flow characteristics during the drawing of coarse ore. With the release of the ore, the flow occurs continually, causing the fine moraine to converge in the ore layer and providing favorable material source conditions for the formation of mud inclusions.
2. The atmospheric rainfall and surface runoff in the mining area flow into the subsidence pit and continuously penetrate underground. When the water is mixed with fine moraine accumulated through a cross-flow, fluid mud inclusions are easily formed in the caving ore bed.
3. The movement behavior of moraine mud inclusions in the caving ore bed was analyzed. A generalized model of the formation mechanism of mud-inclusion-type underground debris flows was established. The model was used to determine the formation mechanism of the underground mud-inclusion type debris flow. Finally, according to the equivalent inverse flux transient electromagnetic method, the accuracy and reliability of the formation mechanism were verified through geophysical prospecting.

## Data Availability

The basic data supporting the research results are all in the article.
